# Time of Application of Sodium Ascorbate on Bonding to Bleached Dentin

**DOI:** 10.1155/2017/6074253

**Published:** 2017-08-13

**Authors:** Kyoung-Hwa Jung, Eun-Mi Seon, An-Na Choi, Yong-Hoon Kwon, Sung-Ae Son, Jeong-Kil Park

**Affiliations:** ^1^Biomedical Research Institute, Pusan National University Hospital, Busan, Republic of Korea; ^2^Department of Conservative Dentistry, School of Dentistry, Pusan National University, Dental Research Institute, Yangsan, Republic of Korea; ^3^Department of Dental Materials, School of Dentistry, Pusan National University, Yangsan, Republic of Korea

## Abstract

This study examined the effects of different application times of sodium ascorbate (SA) on the bond strength of composite resin to bleached dentin. Specimens with an exposed dentin surface were divided into 3 groups according to the type of bleaching agent used: Group A, mixture of sodium perborate (SP) and distilled water (DW); Group B, mixture of SP and hydrogen peroxide (HP); control group, no bleaching. Each group was classified into 10 subgroups. Subgroups IB and DB underwent immediate bonding and delayed bonding, respectively. 10% SA was applied to 3, 5, 10, and 30 minutes and 1, 24, 48, and 72 hours, respectively. Microtensile bond strength (*μ*TBS) was measured after restoration, and the data was analyzed by one-way ANOVA and Scheffé's test. Before restoration, the dentin surfaces were examined by scanning electron microscopy (SEM) and energy dispersive X-ray spectroscopy (EDS). SEM showed that most dentin surfaces were filled with crystals when SA was applied to more than 24 hours. EDS revealed peaks of calcium, carbon, oxygen, and sodium. The application of SA for 5 minutes to 48 hours or for 30 minutes to 24 hours is suggested when a mixture of SP and DW or HP is used, respectively.

## 1. Introduction

The discoloration of the teeth, particularly the anterior teeth, is a serious problem in esthetic restorative dentistry. Tooth bleaching is the most conservative and cost-effective way of improving the appearance of discolored teeth [1].

The intracoronal bleaching technique is the most commonly used technique for endodontically treated teeth [2]. The first description of the intracoronal bleaching technique using a mixture of sodium perborate (SP) and distilled water (DW) was in a congress report by Marsh and published by Salvas [3]. Nutting and Poe used 30% hydrogen peroxide (HP) instead of water in a mixture of SP and water to enhance the bleaching efficacy of the mixture [4].

Although the intracoronal bleaching technique produces an esthetically satisfactory outcome on nonvital teeth, some authors have reported the adverse effects of this technique, such as the reduction of enamel and dentin microhardness [5], external cervical root resorption [6], and a decrease in bond strength between restorative material and tooth substrate [7].

With regard to a decrease in bond strength, some studies have proposed that remnants of peroxide or oxygen may be responsible for inhibiting the polymerization of composite resin [8, 9]. Therefore, it is recommended that the application of composite resin to a bleached tooth surface should be delayed to reduce the adverse effects of bleaching agents on adhesion [10, 11].

Other studies have suggested that the application of a 10% sodium ascorbate (SA) solution as an antioxidant is an effective choice for increasing the bond strength [8, 12–14]. On the other hand, the application time of SA in those studies ranged from one minute to six hours [8, 12, 14]; the optimal application time has not been investigated sufficiently. Therefore, the purpose of this study was to evaluate the effect of different application times of 10% SA after an intracoronal bleaching technique with SP mixed with DW or 30% HP on bonding between the composite resin and dentin and to investigate the optimal application time of 10% SA for improving the bond strength of the composite resin to bleached dentin.

## 2. Materials and Methods

### 2.1. Tooth Preparation

The Institutional Review Board of Pusan National University Dental Hospital (IRB, PNUDH-2014-028) previously approved forty-two caries-free human third molars, and they were used. The teeth were cleaned of soft tissue debris using a manual periodontal curette and stored in 0.5% chloramine-T solution until the beginning of the study. The teeth were ground on a laboratory trimmer to expose the dentin surface under water cooling. The part of the teeth containing the roots was embedded in acrylic resin (Tokuso Curefast; Tokuyama, Tokyo, Japan), and the surfaces were ground with a 600 grit silicon carbide paper under running water to gain smooth and flat dentin surfaces.

### 2.2. Specimen Preparation

The teeth were divided randomly into a control group and two experimental groups according to the bleaching agent type used: Group A, a mixture of 2 g of SP powder and 1 ml of DW, and Group B, a mixture of 2 g of SP powder and 1 ml of 30% HP. Each bleaching agent was applied to the dentin surface for 7 days at 100% relative humidity. The specimens in the control group were stored in DW for 7 days. During this period, the DW was changed every 24 hours. After the specimens were rinsed thoroughly with DW for 30 seconds following the bleaching procedure, experimental groups were sorted into 20 subgroups and control group. For subgroup IB (immediate bonding), the bonding procedure was conducted immediately after bleaching. The specimens in subgroup DB (delayed bonding) were stored in DW for 14 days prior to bonding. The specimens in subgroups SA 3 m, SA 5 m, SA 10 m, SA 30 m, SA 1 h, SA 24 h, SA 48 h, and SA 72 h were immersed in a 10% SA solution for 3, 5, 10, and 30 minutes and 1, 24, 48, and 72 hours, respectively (Table 1). To prepare the 10% SA solution, sodium ascorbate [L(+) ascorbic acid sodium salt] was dissolved and mixed in DW at room temperature. After the storage in 10% SA solution, the dentin surface was cleaned thoroughly with running DW for 30 seconds and dried lightly with compressed air.

### 2.3. Bonding Procedure

For the bonding procedures, Clearfil SE Bond (Kuraray, Osaka, Japan) was applied to the exposed dentin surfaces according to the manufacturer's instructions. The primer was applied to the surface for 20 seconds and evaporated with light air flow. The bond was applied, gently air dried, and cured for 10 seconds using a light-emitting diode- (LED-) visible light curing unit (Elipar S10; 3 M ESPE, Seefeld, Germany). Dual-cure composite core materials (LuxaCore Automix Dual; DMG, Hamburg, Germany) with A3 shade were used for a resin buildup (approximately 4 mm height) and cured for 40 seconds using the same light curing unit. The bonded teeth were stored in 24 ± 1°C DW for 24 hours before sectioning into stick specimens.

### 2.4. Microtensile Bond Strength (*μ*TBS) Test

The stored teeth in DW for 24 hours were sectioned longitudinally to obtain 1.0 × 1.0 mm sticks using a diamond-saw under copious amounts of water. Each subgroup had 10 specimen sticks with a 1 mm^2^ cross section. Each stick was attached individually to a specially designed jig of a microtensile testing machine (Bisco, Schaumburg, IL, USA) using cyanoacrylate cement (Zapit; Dental Ventures of America, Corona, CA, USA) and loaded to failure under the cross-head speed of 1.0 mm/min. The load of fracture was recorded in Newtons (N). To convert the force to MPa, the value was divided by the cross section area (1 mm^2^).

### 2.5. Scanning Electron Microscopy (SEM) and Energy Dispersive X-Ray Spectroscopy (EDS) Evaluation

To observe and evaluate surface condition changes after immersion in 10% SA solution, specimens were prepared. After that specimen surfaces were observed using SEM (JSM-6480LV, JEOL, Tokyo, Japan). The elemental composition of the dentin surface was evaluated by EDS (INCA 7573, Oxford Instruments, Abingdon, UK).

### 2.6. Statistical Analysis

Statistical analysis of the *μ*TBS among subgroups was performed using a one-way ANOVA for test groups, respectively. A post hoc Scheffé's test was followed for a multiple comparison. SPSS 15.0 (SPSS, Chicago, IL, USA) was used for analysis and the significance level was set to *p* < 0.05.

## 3. Results

### 3.1. *μ*TBS

Tables 2 and 3 list the mean *μ*TBS and standard deviation (SD) of all subgroups.

In Group A, the bond strengths of subgroups IB, SA 3 m, and SA 72 h were significantly lower than the other subgroups (*p* < 0.05). No significant difference was observed among the control group and subgroups DB, SA 5 m, SA 10 m, SA 30 m, SA 1 h, SA 24 h, and SA 48 h (*p* > 0.05).

In Group B, the bond strengths of subgroups IB, SA 3 m, SA 5 m, SA 10 m, SA 48 h, and SA 72 h were significantly lower than the control group and subgroup DB (*p* < 0.05), with no significant differences observed among the IB, SA 3 m, SA 5 m, SA 10 m, SA 48 h, and SA 72 h subgroups (*p* > 0.05). In addition, the bond strengths of subgroups SA 30 m, SA 1 h, and SA 24 h had no significant differences compared with those of subgroups SA 10 m, SA 48 h, and SA 72 h (*p* > 0.05).

### 3.2. SEM and EDS

**Figure 1:**
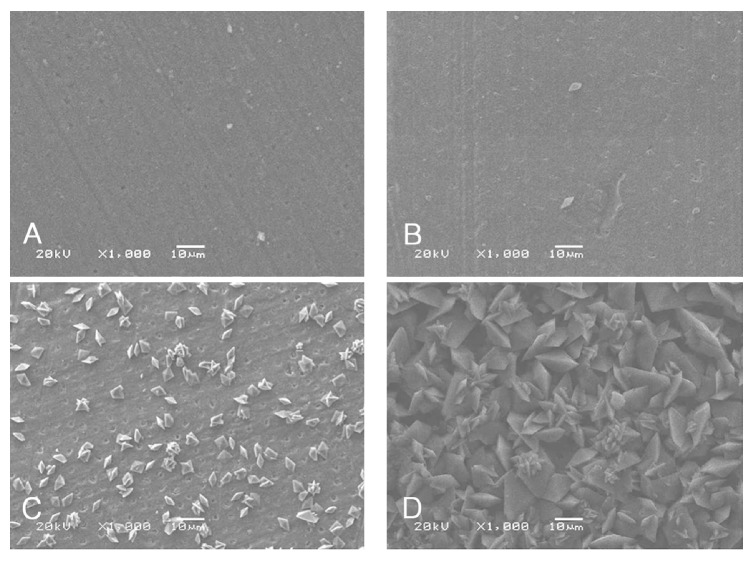
Appearances of dentin surface according to application times of 10% sodium ascorbate (SA) solution after bleaching procedure using scanning electron microscopy (SEM) in the Group A. (A) Immediately after bleaching procedure; (B) application of the 10% SA solution for 30 minutes after bleaching procedure; (C) application of the 10% SA solution for 24 hours after bleaching procedure; (D) application of the 10% SA solution for 72 hours after bleaching procedure.

**Figure 2:**
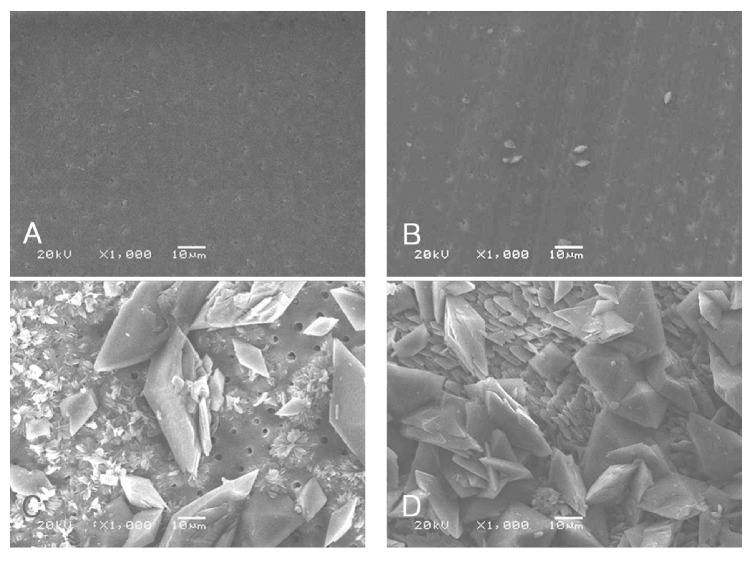
Appearances of dentin surface according to application times of 10% sodium ascorbate (SA) solution after bleaching procedure using scanning electron microscopy (SEM) in the Group B. (A) Immediately after bleaching procedure; (B) application of the 10% SA solution for 30 minutes after bleaching procedure; (C) application of the 10% SA solution for 24 hours after bleaching procedure; (D) application of the 10% SA solution for 72 hours after bleaching procedure.

Figures 1 and 2 present the SEM images of the specimens.

In Group A, there were no visible changes in the dentin surface during first 30 minutes application of 10% SA solution in comparison to the dentin surface of no application of the 10% SA solution (Figures 1(A) and 1(B)). After an application time of 24 hours, the dentin surface was partially covered with crystals (Figure 1(C)). The portion of the dentin surface filled with the crystals and the size of crystal increased in the dentin surface after 72 hours of application time of 10% SA solution (Figure 1(D)).

In Group B, the dentin surface also showed the typical pattern when the 10% SA solution was applied to less than 30 minutes (Figures 2(A) and 2(B)), and most of the dentin surface was also filled with crystals and the crystals grew in size with increasing application time of the 10% SA solution (Figures 2(C) and 2(D)).

**Figure 3:**
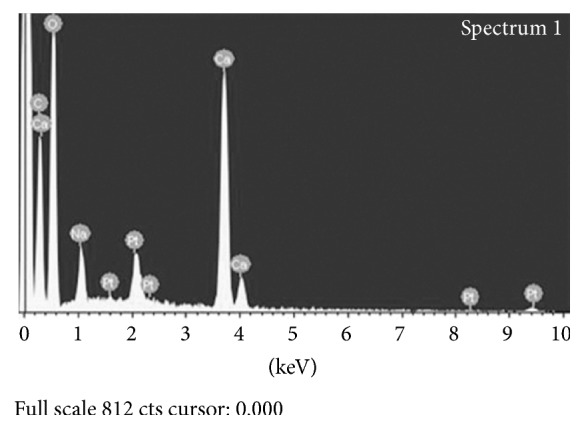
Energy dispersive X-ray spectroscopy (EDS) spectrum. Peaks of calcium (Ca), carbon (C), oxygen (O), sodium (Na), and platinum (Pt) were observed for the crystal on dentin surface.

Figure 3 shows the EDS spectrum of the crystals on the dentin surface. The peaks for calcium, carbon, oxygen, sodium, and platinum were observed.

## 4. Discussion

After intracoronal bleaching procedures, the appropriate adhesion between the tooth structure and intracoronal restorative material is important for withstanding the masticatory forces and ultimately maintaining an esthetic restoration. Several studies revealed the adverse effects of bleaching materials on the marginal seal at the tooth-restoration interface as well as their interference with the bonding of the composite resin to the tooth structures [7–9, 13].

SP is an oxidizer in powder form that is stable under dry conditions but breaks down to sodium metaborate, HP, and nascent oxygen in the presence of acid or water [15]. HP is used most commonly as a bleaching agent and its degradation releases oxygen free radicals or the perhydroxyl anion [16]. Some studies have suggested that the presence of residual oxygen may be due to the decrease in bond strength [8, 17–19]. Torneck et al. and Rueggeberg and Margeson reported that oxygen free radicals could either interrupt the penetration of resin components into the enamel and dentin or disrupt the polymerization of resin [17, 19]. Toko and Hisamitsu suggested that the decrease in bond strength may be caused by the elimination of the nonfibrous organic substrate within the tooth substance after the application of HP [9]. In this study, the bond strength of the composite resin to bleached dentin also decreased after the bleaching process, which is consistent with the results of previous studies [8, 9, 17–19].

Some studies reported that delayed implementation of the restoration process allowed a decrease in the amount of the residual oxygen radicals, which could improve the bond strength at the tooth-restoration interface [10, 11, 20]. No significant differences between the control group and subgroup DB were also found in both groups in the present study.

Another suggestion for increasing the bond strength was the use of antioxidants. Some authors suggested that the application of a 10% SA solution as an antioxidant improves the bond strength of the bleached tooth surface [8, 12–14]. Ascorbic acid and its salts are used as effective antioxidants to reduce various oxides [21]. Lai et al. assumed that the change in the oxidation-reduction potential of the oxidized tooth substance was recovered by SA, which enabled the bonding agents to maintain free radical polymerization and finally regain the reduced bond strength to bleached dentin [8].

In this study, when a 10% SA solution was applied to the bleached dentin for 5 minutes to 48 hours in the Group A and for 30 minutes to 24 hours in the Group B, the bond strengths showed no statistically significant differences compared with those of the control group. In Group A, the application of a 10% SA solution for 3 minutes could not recover the bond strength between the composite resin and bleached dentin surface. In addition, in Group B, the bond strengths were lower than that of the control group when a 10% SA solution was applied to the bleached dentin for 3, 5, and 10 minutes. These outcomes suggest that it may take some time for the 10% SA solution to improve the bond strength between the composite resin and bleached dentin surface. The present results suggest that when a mixture of the SP and DW or a mixture of SP and 30% HP is used as a bleaching agent, the appropriate application time of a 10% SA solution before the bonding procedures are 5 minutes and 30 minutes, respectively.

These results show that the proper application times of a 10% SA solution vary according to the bleaching agents applied. This is probably because of the difference in the amount of HP contained in bleaching agents used. Haywood reported that SP releases less HP and has a slower reaction speed [2]. This might explain why Group B requires a longer application time of a 10% SA solution than Group A.

An interesting finding of this study is that the bond strength decreased when the application time of a 10% SA solution was extended excessively. In Group A, when a 10% SA solution was applied to 72 hours, the bond strength decreased significantly and showed no significant difference compared to the bond strength of subgroup IB. Similarly, in Group B, there was no significant difference between the bond strength of Group IB and those of the groups applied with the 10% SA solution for more than 48 hours.

In this study, SEM revealed crystals on the dentin surface. In addition, as the application time of the 10% SA solution increased, the crystals showed a tendency to increase in size and quantity. After application for more than 24 hours, most of the dentin surface in both groups was covered with crystals. The crystals still remained on the dentin surfaces despite the washing dentin surface with running water for 30 seconds before the bonding procedures. The presence of the crystals at tooth-restoration interface may be responsible for a decrease in the bond strength between the composite resin and bleached dentin.

EDS used in this study is a useful technique for identifying the chemistry of bulk substances and individual particles [22–24]. EDS is used for elemental analysis or chemical characterization of a specimen and can identify and characterize the chemical elements present in deposits semiquantitatively [25]. The EDS spectrum in this study suggested that the crystals on the dentin surface were composed of calcium, carbon, oxygen, platinum, and sodium. Some authors reported that preexisting porosity on the tooth surface was increased by a bleaching treatment [26–28]. McCraken and Haywood detected calcium loss from the teeth exposed to a bleaching agent [29]. Carbon, oxygen, and sodium are elements of SA (C_6_H_7_NaO_6_). In addition, the detection of platinum may be due to the platinum coating procedure conducted during specimen preparation for the SEM evaluation. The experimental results showed that the portion of the dentin surface filled with crystals increased with increasing application time of the 10% SA solution. Therefore, the crystals generated on the dentin surface are probably an outcome of a certain chemical reaction between the SA solution used as an antioxidant and the calcium ions eluted from the porous tooth surface by residual bleaching agent following bleaching procedure.

Overall, when a mixture of SP and DW or SP and 30% HP is used as an intracoronal bleaching agent, it can improve the bond strength of composite resin to bleached dentin with the application of a 10% SA solution on the bleached dentin surface for 5 minutes to 48 hours or for 30 minutes to 24 hours, respectively. The excessively extended application of a 10% SA solution produced crystals on the dentin surface, which might interfere with the bonding between the dentin surface and composite resin.

## 5. Conclusions

Within the limits of this study, the following conclusions can be drawn.

The application of SA for 5 minutes to 48 hours or for 30 minutes to 24 hours is suggested when a mixture of SP and DW or HP is used, respectively. The excessively extended application of a 10% SA solution produced crystals on the dentin surface, which might interfere with the bonding between the dentin surface and composite resin.

## Figures and Tables

**Table 1:** The experimental groups and subgroups used in this study.

Groups		Timing of bonding procedures
Group A (mixture of SP & DW)/Group B (mixture of SP & 30% HP)	IB	Immediate bonding
	DB	Delayed bonding after 14 days
	SA 3 m	After application of SA for 3 minutes
	SA 5 m	After application of SA for 5 minutes
	SA 10 m	After application of SA for 10 minutes
	SA 30 m	After application of SA for 30 minutes
	SA 1 h	After application of SA for 1 hour
	SA 24 h	After application of SA for 24 hours
	SA 48 h	After application of SA for 48 hours
	SA 72 h	After application of SA for 72 hours

Control group,	Immersion in DW for 7 days and then bonding	
no bleaching		

SP: sodium perborate, DW: distilled water, HP: hydrogen peroxide, and SA: sodium ascorbate.

**Table 2:** Mean microtensile bond strength (*µ*TBS) values and standard deviation (SD) of Group A.

Subgroups	Mean ± SD (MPa)
IB	8.02 ± 2.61^A^
DB	23.06 ± 2.96^B^
SA 3 m	8.67 ± 2.21^A^
SA 5 m	16.47 ± 2.94^B^
SA 10 m	20.57 ± 4.14^B^
SA 30 m	20.59 ± 4.43^B^
SA 1 h	22.38 ± 4.05^B^
SA 24 h	17.91 ± 4.37^B^
SA 48 h	16.58 ± 3.59^B^
SA 72 h	9.18 ± 1.99^A^
Control group	22.67 ± 3.77^B^

Different superscript letters in column indicate statistically significantly different groups (*p* < 0.05). IB: immediately bonding; DB: delayed bonding. Subgroup SA 3 m, SA 5 m, SA 10 m, SA 30 m, SA 1 h, SA 24 h, SA 48 h, and SA 72 h were applied to 10% sodium ascorbate (SA) solution for 3, 5, 10, and 30 minutes and 1, 24, 48, and 72 hours, respectively, before bonding procedure.

**Table 3:** Mean microtensile bond strength (*µ*TBS) values and standard deviation (SD) of Group B.

Subgroups	Mean ± SD (MPa)
IB	7.93 ± 2.06^a^
DB	22.86 ± 4.07^c^
SA 3 m	8.02 ± 2.26^a^
SA 5 m	9.29 ± 2.31^a^
SA 10 m	14.29 ± 4.38^ab^
SA 30 m	20.94 ± 3.50^bc^
SA 1 h	21.29 ± 3.62^bc^
SA 24 h	20.84 ± 3.63^bc^
SA 48 h	14.40 ± 4.33^ab^
SA 72 h	14.35 ± 4.56^ab^
Control group	22.67 ± 3.77^c^

Different superscript letters in column indicate statistically significantly different groups (*p* < 0.05). IB: immediately bonding; DB: delayed bonding. Subgroup SA 3 m, SA 5 m, SA 10 m, SA 30 m, SA 1 h, SA 24 h, SA 48 h, and SA 72 h were applied to 10% sodium ascorbate (SA) solution for 3, 5, 10, and 30 minutes and 1, 24, 48, and 72 hours, respectively, before bonding procedure.
